# Influence of occlusal reduction design on the fracture resistance and biomechanical behavior of endocrowns restoring maxillary premolars

**DOI:** 10.1186/s12903-023-03688-3

**Published:** 2024-01-19

**Authors:** Mahy Hassouna Abbas, Fatma Abdallah Elerian, Abdallah Ahmed Elsherbiny, Nesma Mohamed Magdy Elgohary, Abeer Atout

**Affiliations:** 1 Fixed Prosthodontics Dept, Faculty of Dentistry, Mansoura University and Delta University for Science and Technology, Mansoura, Egypt; 2 Department of Mechanical Engineering, College of Engineering, University of Bisha, P.O. Box 001, Bisha, 67714 Saudi Arabia; 3 Mansoura University, Mansoura, Egypt; 4 Production Engineering and Mechanical Design Department, Faculty of Engineering, Mansoura University, Mansoura, Egypt; 5 Fixed Prosthodontics Dept, Faculty of Dentistry, Mansoura University, Mansoura, Egypt; 6 Department of prosthetic dental sciences , College of Dentistry, Jazan University, KSA and Fixed Prosthodontics Dept, Faculty of Dentistry, Mansoura University, Mansoura, Egypt

**Keywords:** Endocrowns, Endodontically-treated teeth, Ceramic material, Finite element analysis

## Abstract

**Purpose:**

To investigate the effect of different occlusal reduction design on stress distribution and fracture resistance of different endocrown systems.

**Material and methods:**

Sixty-four maxillary human premolars with endodontic treatment, prepared for endocrowns were divided into 2 groups (*n* = 32) according to the occlusal design: Butt joint preparation (B group) and Anatomical preparation (A group). Each group were subdivided into four groups according to ceramic systems: IPS E max CAD (EM group), monolithic zirconia (ZR group), Nacera Hyprid (NH group) and PEKKTON (PE group). After manufacturing of endocrowns and adhesive bonding the specimens were thermomechanically loaded and subsequently they were tested in a universal testing machine for evaluating the fracture resistance. The specimens failure mode was qualitatively assessed. The stress distribution in each group was assessed using three-dimensional finite element analysis (FEA). 1-way ANOVA and the Post Hoc Tukey HSD test were used to evaluate the data (a = .05).

**Results:**

The fracture resistance values between the groups showed statistically significant variations. The B PE and A PE groups had a higher ratio of fracture resistance values. Regarding failure mode, ceramic endocrowns recorded mainly irreparable failures. FEA showed that anatomical occlusal preparation have reduced the stress concentration under all endocrown systems.

**Conclusion:**

Endocrowns could be used to restore endodontically treated maxillary premolars. PEKKTON endocrowns with anatomical preparations revealed most appropriate restoration. The tested new endocrown systems enhanced the biomechanical performance of the tooth.

**Clinical significance:**

The innovative endocrown systems (PEKK, Nacera Hyprid) can be seen as a promising choice for restoration of severely-destructed endodontic treated premolars, with less stress transmit to the residual tooth structure. Although the traditional endocrown technology might increase the longevity of tooth bonding, it shouldn’t be used for clenching cases since the risk of failure is too great overall.

## Background

Due to tooth structural loss from dental trauma or cavities, endodontically treated teeth are highly susceptible to biomechanical failure during restoration [1]. Therefore, it is crucial to choose the best restorative material and protect the remaining tooth structure to ensure long-term success [2]. Traditionally, a crown with a retained post and core has been used for restoration. However, this approach may compromise the tooth’s ability to resist mechanical forces and increase the risk of root fracture [3]. With the advancement of modern dental restoration techniques, the concept of “minimally invasive” dentistry has gained popularity. Practitioners are now more interested in selecting the optimal repair technique.

Endocrowns have emerged as a significant method for tooth restoration following endodontic treatment. This is due to improvements in bonding with the tooth and the concept of minimally invasive dentistry [4]. An endocrown consists of a central retainer placed inside the pulp chamber and a crown portion to restore the tooth coronally [4, 5]. It provides both macromechanical retention, which is supplied by the walls of the pulp chamber, and micromechanical retention, which relies on adhesive bonding. As a result, the endocrown is considered a monoblock restoration. Endocrown restorations offer several advantages, including their conservative nature, shorter treatment time, and lower cost. They are particularly suitable for cases involving teeth with anatomical malformations such as varied roots, calcified or curved root canals, and significant coronal structural loss [6]. Endocrowns have been found to exhibit better clinical performance compared to conventional treatment approaches [6, 7].

Clinical research has shown no significant difference in survival rates between molars restored with endocrowns and those restored using conventional methods [6]. However, it should be noted that endocrown-restored premolars tend to have lower clinical performance compared to endocrown-restored molars [7]. The primary cause of failure in premolars restored with endocrowns is cohesive failure of bonding [8]. Furthermore, premolars have a higher leverage compared to molars due to the relationship between the crown base and height [9]. This results in more horizontally (non-axial) directed forces being applied, which could potentially impact the fracture resistance of the restoration [10].

The method of preparation for Endocrown restorations differs from traditional crown preparations, as this adhesive restoration does not require a subgingival margin, which can potentially cause gingival inflammation [11]. In the literature, various intrapulpal depths and occlusal preparation schemes have been proposed [12, 13]. Anatomic cusp reduction designs have been shown to increase the resistance to fracture of restorations in endodontically treated maxillary premolars [12].

For the CAD/CAM production of endocrowns, a variety of materials are used, including glass ceramics, lithium silicate ceramics, zirconia-strengthened ceramics, ceramics with polymer infiltration, and resin nanoceramics [14–16]. One of the recently developed materials is monolithic zirconia without veneering porcelain, with yttria-stabilized tetragonal zirconia polycrystal (Y-TZP) being the most common type [17].

The development of a new generation of hybrid blocks for CAD/CAM processing has been made possible through ongoing research into biomimetic materials that closely mimic the physico-mechanical characteristics of natural tooth structure [18]. One such recent addition to the dentistry industry is the Nacera® Hybrid, a brand-new variety of hybrid blocks designed for chairside or labside milling machines [19].

Polyaryletherketones (PAEK) are high-performance thermoplastic polymers that include polyetherketoneketone (PEKK) and polyetheretherketone (PEEK). PEKK, a relatively recent introduction, exhibits excellent compressive strength, which is 80% higher than that of unreinforced PEEK. This enhanced strength contributes to its superior long-term fatigue properties and biocompatibility [20, 21]. PEKK’s mechanical characteristics are comparable to those of natural dentition, promoting a biomechanical fit between the tooth and the restoration and reducing the risk of fracture [22, 23]. The finite element method is an effective dental biomechanical technique for analyzing stress concentration. It takes into account surface geometry, boundary conditions, material physical characteristics, and loading conditions [24–26].

For endodontically treated premolars, there is a lack of clear preparatory recommendations in the literature to ensure optimal biomechanical behavior [7, 8]. Additionally, there is no consensus in the literature regarding the best material for restoring teeth that have undergone endodontic treatment with the most favorable mechanical behavior [13].

Therefore, the aim of this study was to investigate how different CAD/CAM materials and two occlusal preparation designs (horizontal butt reduction and anatomical reduction) affect the fracture resistance and stress distribution of upper premolars that have undergone endodontic treatment and are subsequently restored with an endocrown.

The null hypothesis of this study was that the CAD/CAM materials used and the occlusal reduction designs would not have an impact on the fracture resistance and biomechanical behavior of endocrowns in upper premolars.

## Materials and methods

### Natural teeth collection

This study was fixed at faculty of Dentistry, Mansoura University, Egypt after approval of Research Ethics Committee of Faculty of Oral and Dental Medicine, Delta University for science and technology with number FODMRC-202200114. Sixty-four sound maxillary first premolars of human that was extracted due to periodontal disease or for orthodontic purposes were obtained from the faculty of dentistry at Mansoura and Delta University oral and maxillofacial surgery department and cleaned with a Suprassontm P5 Booster ultrasonic scaler (France). All the teeth were then preserved in a 0.1% Thymol solution (Caelo, Hilden, Germany) at room temperature. Completely developed apices, the lack of carious lesions, lack of line of fracture, and equivalent bucco-lingual (BL) and mesio-distal (MD) dimensions as measured using a digital caliper were the criteria of selection of the teeth. To prevent dehydration, the teeth were saved in distilled water at room temperature throughout all testing durations.

### Sample grouping

Teeth were randomly divided into two groups (*n* = 32) according to the preparation design of the endocrown: Group B; Butt joint occlusal preparation and Group A; Anatomical occlusal preparation. Each group was further sub-divided into two subgroups (*n* = 8) according to the material used (Table 1).

**The tested sub-groups are:**

- B EM; Butt joint occlusal reduction, Emax Cad endocrown.
- B ZR; Butt joint occlusal reduction, Zirconia endocrown.
- B NH; Butt joint occlusal reduction, Nacera Hyprid endocrown.
- B PE; Butt joint occlusal reduction, PEKK endocrown.
- A EM; Anatomical occlusal reduction, Emax CAD endocrown.
- A ZR; Anatomical occlusal reduction, Zirconia endocrown.
- A NH; Anatomical occlusal reduction, Nacera Hyprid endocrown.
- A PE; Anatomical occlusal reduction, PEKK endocrown.

**Table 1:** Materials used in this study

Material	Product name	Composition	Manufacturer
1) Lithium disilicate glass ceramic	IPS e.max CAD (LT A2/C 14	- Main component: SiO2 (57–80 wt%) - Other contents: Li2 O, K2 O, MgO, Al2 O3, P2 O5, ZrO2, ZnO and coloring oxides	Ivoclar Vivadent, Schaan, Liechtenstein
2) Translucent Zirconia	Ceramill Zolid HT+ White ZrO2	partially stabilized with yttrium and enriched with aluminium	(Ceramill Zolid HT, Amman Girrbach, Germany)
3) Nacera Hybrid	Tough, fully polymerized radiopaque composite material with optimized, high-density filler technology (Hybrid A2, Block S)	50% Nano-Glass and 50% Polymer-Matrix	DOCERAM Medical Ceramics GmbH Hesslingsweg 65–67 | D-44309 Dortmund / Germany
4) High performance polymer PEKK	PEKKTON ivory milling blank (98.5/t20mm)	-Polyetherketoneketone (PEKK) 90% -Titanium Dioxide (TiO2) 10%	Cendres + Metaux SA, Biel/Bienne, Switzerland

### Endodontic treatment of all teeth

Endodontic treatment was performed on all teeth using a Ni-Ti rotary file system (Race/25 mm) following the manufacturer’s guidelines [27]. The canals were irrigated with 5.25% sodium hypochlorite liquid [4], followed by the administration of 17% EDTA solution for 5 minutes to remove the smear layer [27]. The root canals were then obturated. After completing the root canal therapy for all teeth, the gutta-percha was removed from the canal entry using a circular bur under a water cooling system. Then, applying a tinny coating of a light-cured dental adhesive (All-Bond Universal). Then, kidney-shaped access canals were filled about 2 mm with a flowable composite resin material (Nexcomp Flow A3 META® BIOMED, Korea) [4, 27, 28]. Following the manufacturer’s recommendations, this bonding agent was inserted inside the cavity for 10–15 seconds, applying air for 10 seconds, then light-cured by an LED light-curing unit (Elipar DeepCure-S), power intensity 1470 mW/cm2 (− 10%/+ 20%)for 10 seconds. All the selected teeth were fixed into a resin block vertically using a dental surveyor (Milling unit BF 2) [4, 29, 30]. To create a layer similar to the periodontal ligament (approximately 0.3 mm), the “Transitional Wax Technique” and a light-body polyvinyl siloxane rubber base impression material were used [22, 30].

### Endocrown preparation

The selected teeth were prepared for endocrown restoration. To ensure standardize the preparation dimensions for all the endodontically treated teeth, a Computerized Numerical Control (CNC) milling machine (C.N.C Premium 4820, imesicore, Eiterfeld, Germany) was used. For the butt joint preparation a central kidney-shaped retention cavity measuring 4 mm in depth, 3 ± 0.2 mm in mesiodistal width, and 5 ± 0.2 mm in buccopalatal width with internal taper of the axial wall 6^°^ and a circular butt-margin measuring 2 mm in diameter, a circular axial wall thickness of 2 ± 0.2 mm [31]. For anatomical preparation, the same endocrown preparation with the occlusal surface was prepared following the anatomical contour of the cusps [32].

### CAD/CAM fabrication of Endocrown restoration

For all the selected teeth in this study, a standard endocrown preparation was performed using CAD/CAM technology. A digital scanner (Ceramill Map 400 scanner, Amann Girrbach) was used to create digital impressions and generate a file in the standard tessellation language (STL). The STL file was then exported to a software program (Ceramill Mind, Amann Girrbach).

To ensure standardized cement space in all endocrowns, a distance of 50 μm [13]. The endocrown restorations were milled using a Ceramill Motion 2 (5x) computer-controlled milling device, utilizing four different types of CAD/CAM materials: Translucent Zirconia (Ceramill Zolid HT, Amann Girrbach, Germany), Lithium disilicate glass ceramic (Ivoclar Vivadent, Schaan, Liechtenstein), Nacera hybrid (DOCERAM Medical Ceramics GmbH, Germany), and high-performance polymer PEKK (PEKKTON ivory milling blank 98.5/t20mm, Switzerland).

For Group PE, sixteen endocrowns were dry-milled using one Pekkton ivory milling blank and sharp, single-bladed, slide-coated milling equipment (CORiTEC). Subsequently, all restorations were cleaned using an ultrasonic cleaner for 3 minutes. The proper fit of the endocrowns was then tested using a sharp probe and an adaption-checking spray (Renfert Occlutec Spray).

### Cementation of Endocrown restoration

Before cementation, all endocrowns underwent surface treatments following manufacturer recommendations. Nacera-hybrid endocrowns were etched for 20 seconds using a brush and 8% hydrofluoric acid gel (Porcelain Etch, DentoBond Porcelain Fix Itena Products, France). The endocrowns were then rinsed under running water for 20 seconds and dried for 30 seconds with moisture-free compressed air. Porcelain silane (DentoBond Porcelain Fix Itena Products, France) was applied to the etched surfaces of the endocrowns using a brush and allowed to dry for 1 minute. For groups PE and EM, the fitting surfaces of the endocrowns were sandblasted for 5 seconds at a distance of 1 mm and an angle of 45° using Zeta Sand and unrecycled 110 μm aluminum oxide under a pressure of 2 bar. The endocrowns were then thoroughly cleaned with steam and dried for 20 seconds with oil-free air [33].

The teeth with endocrown preparations were etched using a 37% phosphoric acid etching gel (N-Etch Etching Gel) for fifteen seconds, followed by gentle drying with air. For cementation, a dental adhesive resin cement (SuperCem, Self-Etch Self-Adhesive) was mixed and applied according to the manufacturer’s guidelines. It was placed on the internal surface of the restoration and then completely seated onto the appropriate tooth. Prior to spot curing, any excess cement was removed using a brush. A weight of one kg was applied over the cemented specimens for five minutes to achieve a standardized homogeneous cement film thickness under equal pressure during cementation. Then, light curing was applied to the margin for 40 seconds in each direction at a distance of 10 mm to all surfaces [34].

### Thermal-cycling, fracture testing and failure analysis

To simulate the intra-oral environment, all samples were artificially aged for 24 hours following the cementation of the endocrowns. Subsequently, they were stored in distilled water at 37 °C in an incubator [13, 27]. The samples were then subjected to thermal cycling using a simulation system (Thermocycler, Robota, Alexandria, Egypt). This involved cycling the samples through 10,000 cycles with temperature changes between 5 °C and 55 °C. Each cycle consisted of a 30-second dwell period in a distilled water bath, with a transfer time of 5 seconds. This simulation aimed to replicate approximately one year of clinical use [35, 36].

After the thermal cycling, the samples were loaded in a universal testing machine (Model 3345; Instron Industrial Products, Norwood, MA, USA) until permanent deformation or failure occurred. A 5 kN load cell and a 6 mm diameter stainless steel ball-shaped loading piston were used to apply a compressive force axially and centrally. The data was recorded using computer software (Instron® BluehillLite Software). The failure mode was then identified and classified into different types. Type I fractures involved slight fractures in the coronal tooth structure or the restoration. Type II fractures indicated cohesive failure of the restoration or significant shattering of the coronal tooth structure. Type III fractures involved cohesive and/or adhesive restorative failures, with root involvement above the level of the bone crest. Type IV fractures were characterized by severe crown and root fractures [37, 38].

### Finite element analysis in three dimensions (3D FEA)

Based on the literature [10, 24, 37–39] and the manufacturer, mechanical properties of the materials were presented in (Table 2). Young’s Modulus measures the stiffness of an elastic material. Poisson’s ratio is the ratio of the transverse strain (perpendicular to the applied load) to the axial strain (in the direction of the applied load) [10].

**Table 2:** Mechanical properties and Weibull moduli of the finite element models’ utilized structures

	Young’s modulus (MPa)	Poisson ratio (V)	Characteristic strength (MPa)	Weibull modulus (𝑚)
E-max Cad	**95,000**	**0.25**	**609.80**	**13.4**
Zirconia	**210,000**	**0.25**	**700**	**–**
Nacera hyprid	**9900**	**–**	**490**	**–**
PEKK	**5100**	**0.36**	**215**	**200**
Resin cement	**6000**	**0.27**	**283.30**	**4.02**
Flowable composite	**7000**	**0.25**	**–**	**–**
Spongious bone	**1370**	**0.3**	**–**	**–**
Cortical bone	**10,700**	**0.3**	**–**	**–**
Enamel	**84,100**	**0.33**	**42.41**	**5.53**
Dentine	**18,600**	**0.32**	**44.45**	**3.35**
Pulp	**–**	**0.45**	**–**	**–**
Periodontal ligament	**68.9**	**0.45**	**–**	**–**
Gutta percha	**0.69**	**0.45**	**–**	**–**
Acrylic resin	**2900**	**0.31**	**80**	**–**

**Fig. 1:**
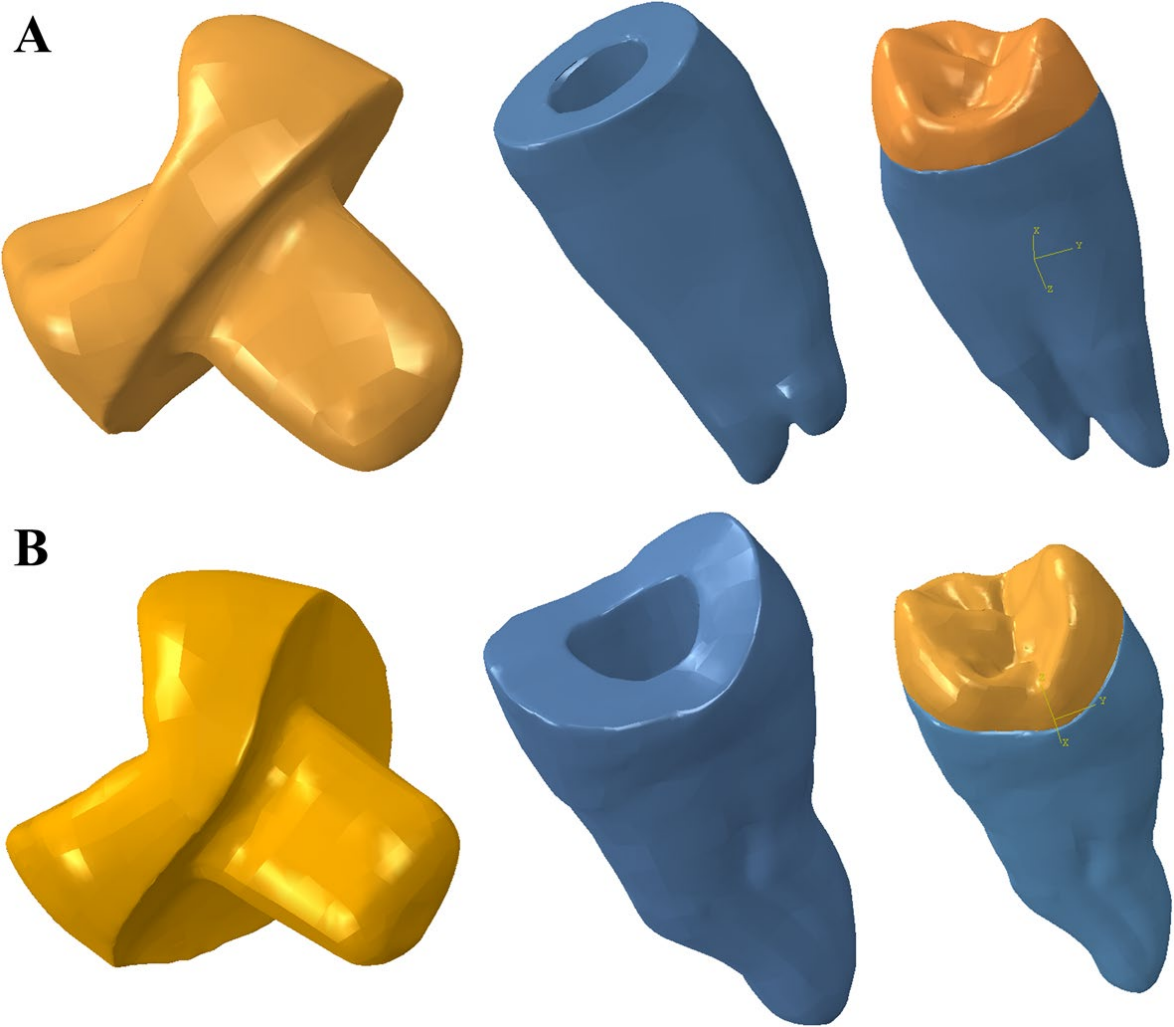
Finite element solid model generation **A** Butt joint preparation model, **B** Anatomical preparation model

To assess the internal structural behavior and stress distribution in the remaining tooth structure (enamel and dentin), endocrown systems, and cement lines under axial load application, a finite element analysis (FEA) method was employed [27]. The process involved creating a finite element model using scanning technology. Initially, the 3D geometry of a recently extracted healthy maxillary first premolar was obtained [40]. The tooth was scanned using a highly sensitive 3D optical scanner (Identica hybrid, Medit Dental, Seoul, Korea) equipped with a blue LED light source and triple camera scanning technology. Specialized software (colLab Scan, v2.0.0.4, Medit Dental, Seoul, Korea) was used for the scanning process, and the data obtained were saved in STL file format. The scanned tooth data was then used to create a 3D solid model using CAD 3D modeling software (SOLIDWORKS® 3D CAD, Dassault Systemes, Ile-de-France, France) [10] (Fig. 1). To simulate the root structure, a 3D acrylic resin cylindrical block with dimensions of 16 mm in diameter and 24 mm in height was developed [38]. The root canal spaces were filled with gutta-percha material, leaving a 5 mm gap from the orifice. A flowable composite base (2 mm thickness) was applied to fill the pulp chamber [10, 41]. Two models were created for the in vitro study: one for the butt joint group (Group B) and one for the anatomical group (Group A). For each group, four models were created for each endocrown material: Model B EM, Model B ZR, Model B NH, and Model B PE for the butt joint preparation, and Model A EM, Model A ZR, Model A NH, and Model A PE for the anatomical preparation [42].

**Fig. 2:**
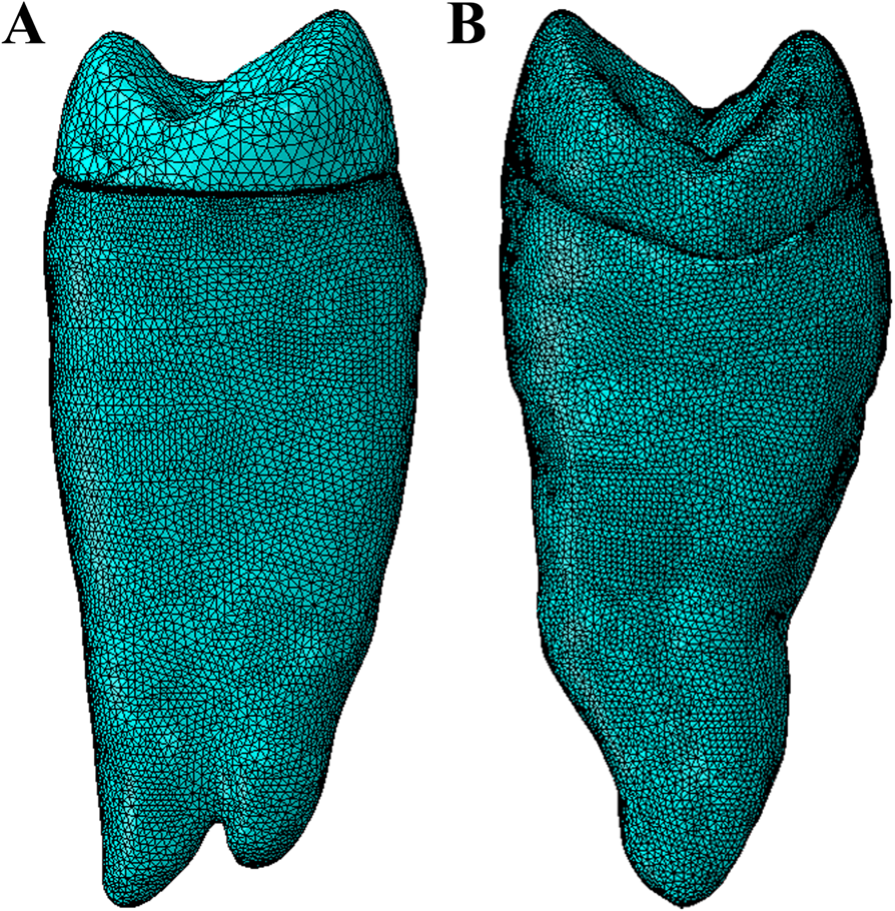
Mesh design **A** Anatomical design, **B** Butt joint design

The finite element mesh was generated using a FEA program (Abaqus, 3DEXPERIENCE R2019x®, Dassault Systemes Simulia Corp, Providence, RI, USA). Linear tetrahedral elements of type C3D4 were used to obtain the geometric 3D solid models for all endocrown systems [27, 41, 43]. The anatomical endocrown model consisted of approximately 530,687 elements and 753,889 nodes, while the butt joint endocrown model had around 308,819 elements and 725,394 nodes (Fig. 2). The software defined the tooth structure, root length, restored the cancellous bone to 0.7 mm, and created a periodontal ligament gap of 0.3 mm around the teeth. The cement space was set at 50 μm. The model structures were assumed to be homogeneous, isotropic, and linearly elastic.

A static compressive load was applied axially and centrally using a spherical solid rigid material (SSRM) with a diameter of 6 mm, a load cell with a force of 5 kN, and a crosshead speed of 0.5 mm/min. Prior to the analysis, the mechanical properties of the materials, boundary conditions, loading angle, and element arrangement were determined. A structural linear static analysis was performed to determine the distribution of stress in the critical region. The equivalent stresses (Modified von Mises, mvM), which are considered more representative of multiaxial stresses, were analyzed in megapascals (MPa) for the premolar tooth, cement layer, and restorative materials. The results were visualized using a linear color scale, with blue indicating the lowest stress values and red and light orange indicating the highest stress values for all models.

Data analysis was performed by SPSS software, version 25 (SPSS Inc., PASW statistics for windows version 25. Chicago: SPSS Inc.). Qualitative data were described using number and percent. Quantitative data were described using mean ± Standard deviation for normally distributed data after testing normality using Shapiro Wilk test. Significance of the obtained results was judged at the (≤0.05) level.

## Results

**Fig. 3:**
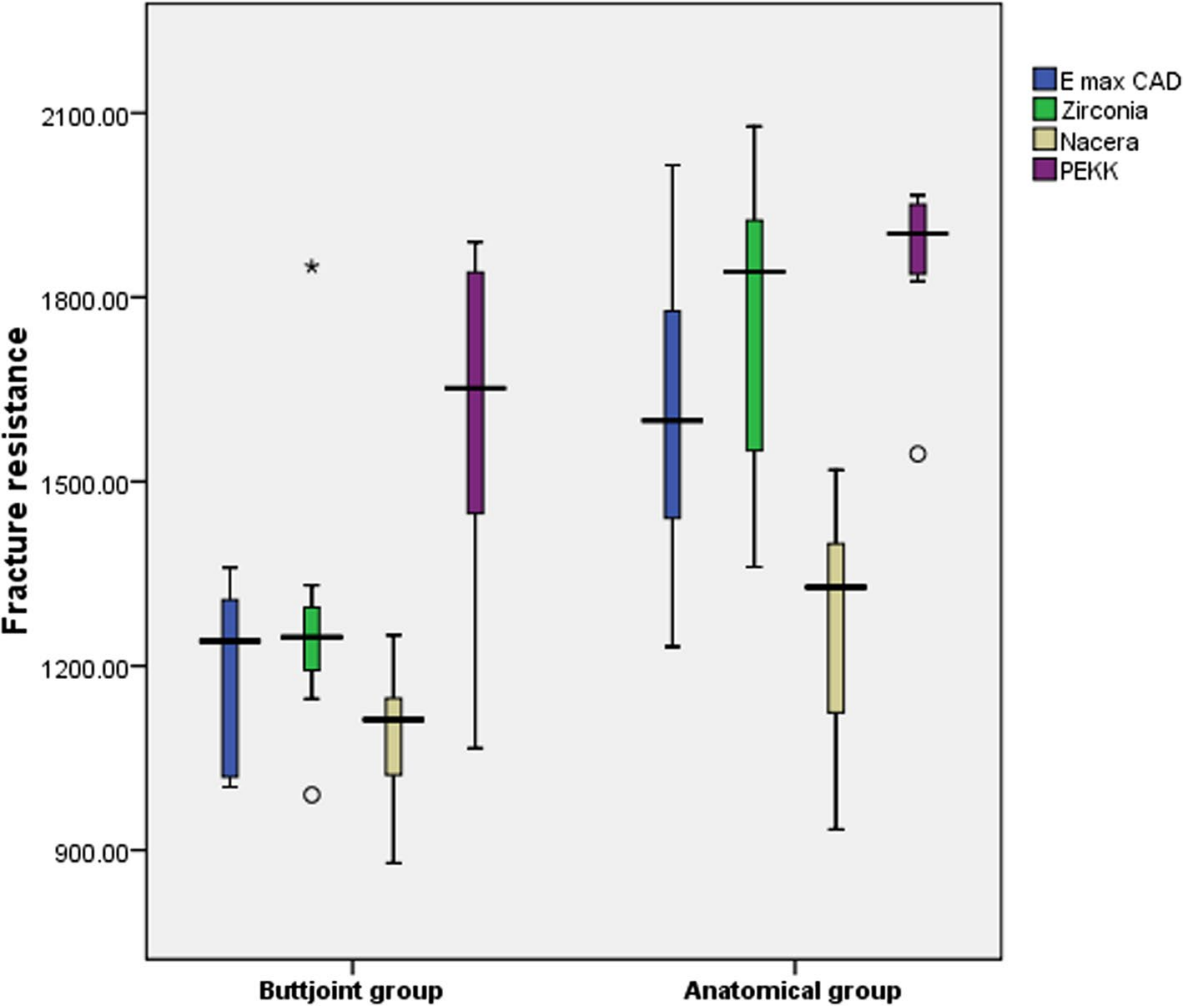
Box plot showing fracture resistance (N) in all samples

There was no failure of all the tested specimens after the artificial thermocycling aging (100% survival rate for all groups). There was a significant difference statistically among the restorative materials shown by One-way ANOVA within the Butt joint group, (F = 5.37; *P* = 0.005), and anatomical group (F = 11.54; *P* < 0.001). The Tukey HSD test revealed that the A PE group had the highest fracture resistance values (1862.0 ± 137.35), statistically different from the other groups. Student t-test was used to assess the effect of the occlusal reduction on the resistance of fracture between the endocrown systems. It showed a significant difference between the two preparation designs within all endocrowns (Table 3, Fig. 3).

**Table 3:** Descriptive statistics and intergroup comparison for fracture resistance (N)

	E max CAD	Zirconia	Nacera Hyprid	PEKK	One Way ANOVA test
Butt joint group	**1187.13 ± 150.83**^**a**^	**1288.63 ± 248.79**^**b**^	**1086.63 ± 114.26**^**c**^	**1604.63 ± 291.61**^**abc**^	***F*** **= 5.37** ***P*** = 0.005*
Anatomical group	**1610.13 ± 248.35**^**ad**^	**1759.13 ± 255.1**^**b**^	**1269.38 ± 209.07**^**bdc**^	**1862.0 ± 137.35**^**ac**^	**F = 11.54** ***P*** < 0.001*
Student test	**t = 4.12** ***p*** = 0.001*	**t = 3.73** ***p*** = 0.002*	**t = 2.17** ***p*** = 0.048*	**t = 2.55** ***p*** = 0.04*	

*statistically significant, similar superscripted letters denote significant difference within same row by Post Hoc Tukey test

Two-way ANOVA test was used to study the effect of occlusal design, restorative material change, and combined effect of ceramic material and occlusal design on fracture resistance. It showed that there was a significant difference between occlusal design (*P* = .001), between ceramic material (*P* = .001), and no significant difference between combined effect of restorative material and occlusal design (*P* = .496) (Table 4).

**Table 4:** Two Way ANOVA test showing effect on fracture resistance

Source	Type III Sum of Squares	df	Mean Square	F	***p*** value
**Corrected Model**	6.609E6^a^	7	944,154.679	10.932	.001*
**Intercept**	1.484E8	1	1.484E8	1718.018	.001*
**Designs**	2,286,144.000	1	2,286,144.000	26.471	.001*
**Materials**	4,113,956.375	3	1,371,318.792	15.878	.001*
Designs * materials	208,982.375	3	69,660.792	.807	.496
**Error**	4,836,444.250	56	86,365.076		
**Total**	1.598E8	64			
**Corrected Total**	1.145E7	63			

a. R Squared = .577 (Adjusted R Squared = .525)

**Fig. 4:**
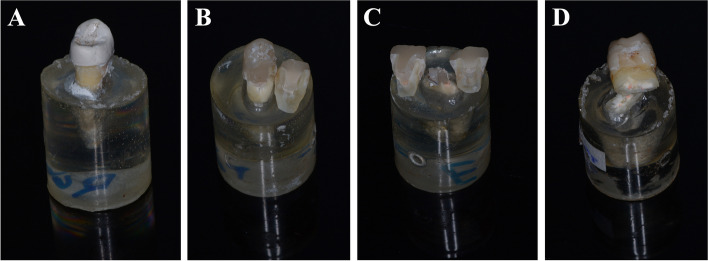
An image showing different fracture patterns; **A** Type I; **B** Type II, **C** Type III and **D** Type IV

All the fractured specimens were photographed with high quality digital camera to evaluate different modes of failure. Within all the tested groups the most common mode of failure was represented as percentage within (Table 5) and shown in (Fig. 4).

**Table 5:** Classification of the failure mode

	Type I	Type II	Type III	Type IV
Emax CAD	0	0	5 (62.5%)	3 (37.5%)
Nacera hybrid	2 (25%)	4 (50%)	2 (25%)	0
Zirconia	0	0	2 (25%)	6 (75%)
PEKK	4 (50%)	3 (37.5%)	1 (12.5)	0

The (mvM) stresses in the prosthetic restorations, individual tooth, luting cement and flowable composite are presented in (Table 6).

**Table 6:** Maximum modified von Mises stress (MPa) for tooth, cement lines, flowable composite and restorative materials for all models

Models	Zirconia		Emax CAD		Nacera Hyprid		PEKK	
	B ZR	A ZR	B EM	A EM	B NH	A NH	B PE	A PE
Tooth	28.4	25.4	23.8	19.1	18.1	15.1	12.7	11.1
Cement line	14.1	13.1	19.1	18.1	22.4	21.3	28.1	27.1
Flowable composite	15.1	12.9	12.4	11.1	9.1	8.2	7.5	4.1
Restorative materials	55.8	62.5	52.1	54.1	40.1	47.2	67.1	72.5

### Stress distribution in endocrown materials

**Fig. 5:**
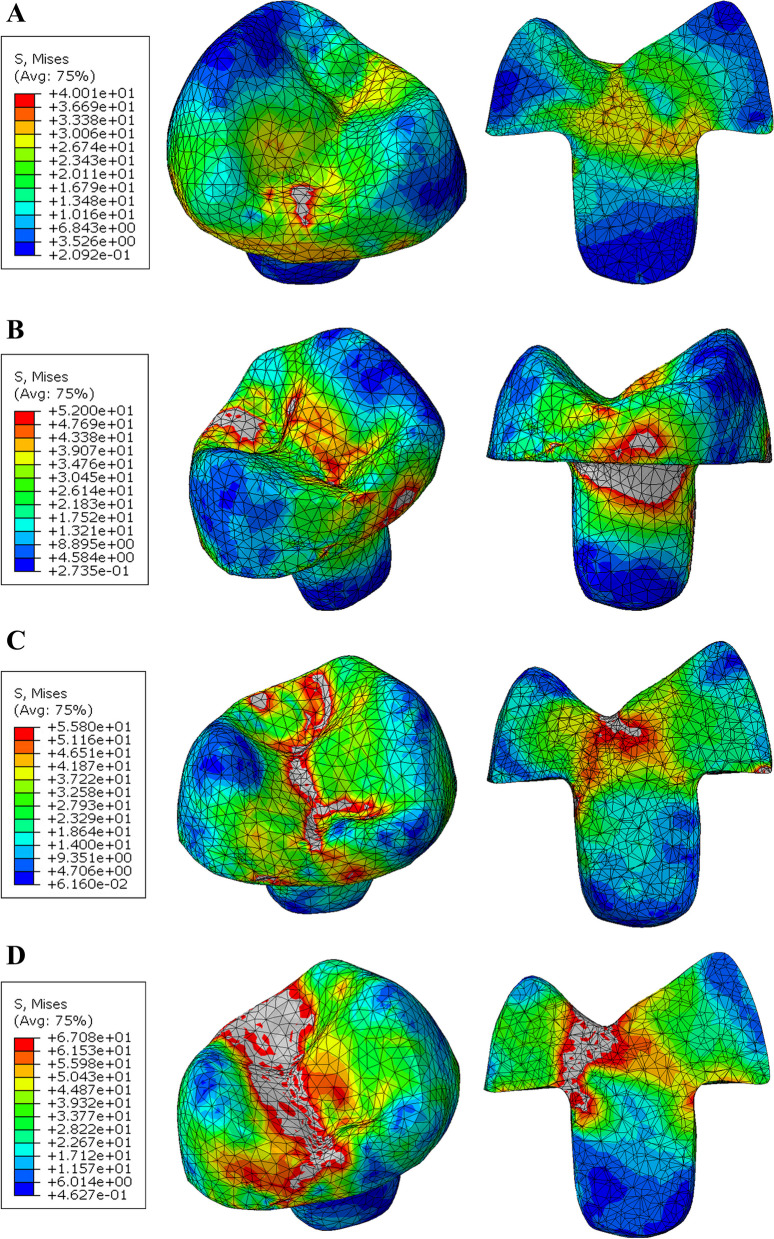
Von Mises stress distributions (MPa) in endocrown restoration (but joint design) **A** Nacera Hyprid **B** Emax CAD **C** Zirconia **D** PEKK

**Fig. 6:**
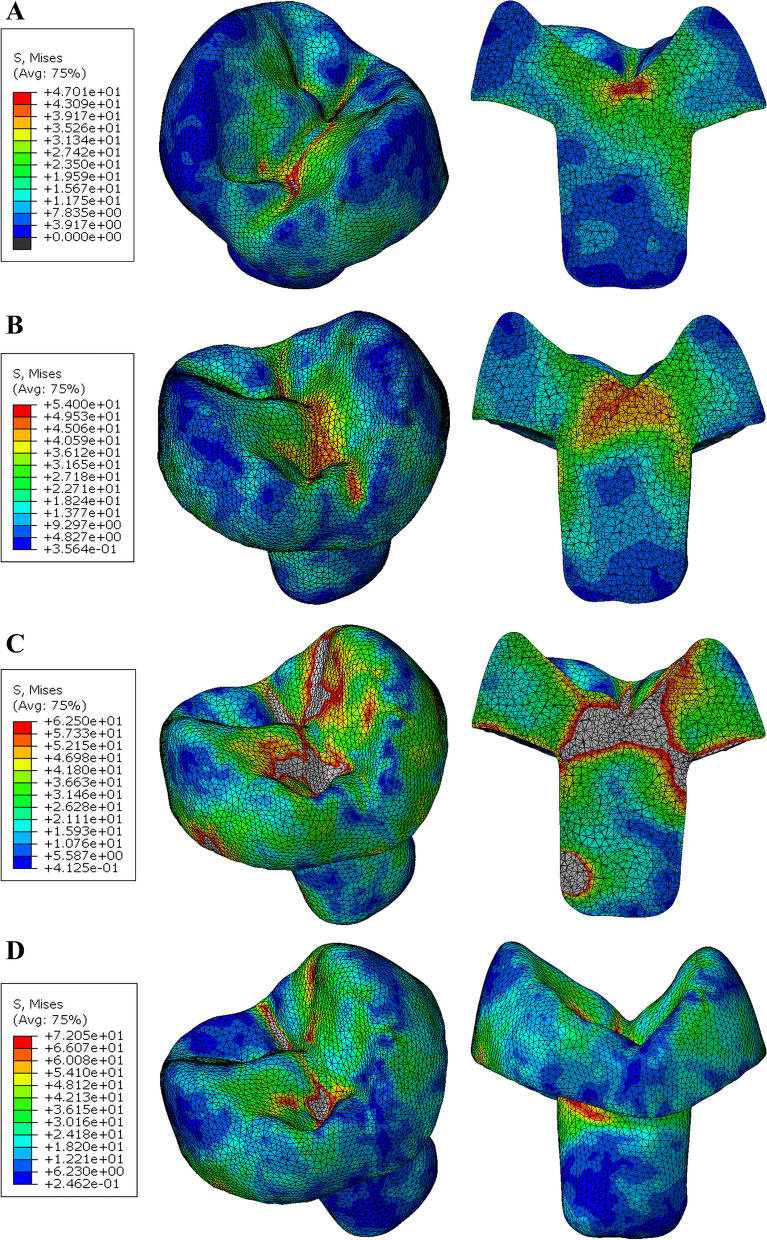
Von Mises stress distributions (MPa) in endocrown restoration (Anatomical design) **A** Nacera Hyprid **B** Emax CAD **C** Zirconia **D** PEKK

In the endocrown restoration, the group restored with Nacera Hyprid with butt joint reduction showed slightly lower mvM stress values (40.1 MPa). In comparison, the PEKK restorations with anatomical reduction presented higher mvM stress values (72.5 MPa). When compared between the two occlusal designs within each material the lowest values of mvM stress were observed in restorations with butt joint occlusal reduction followed by restorations with anatomical butt joint reduction. According to the color map the maximum stresses were appeared to be located at the occlusal surfaces extended at mesial and distal direction (Figs. 5 and 6).

**Fig. 7:**
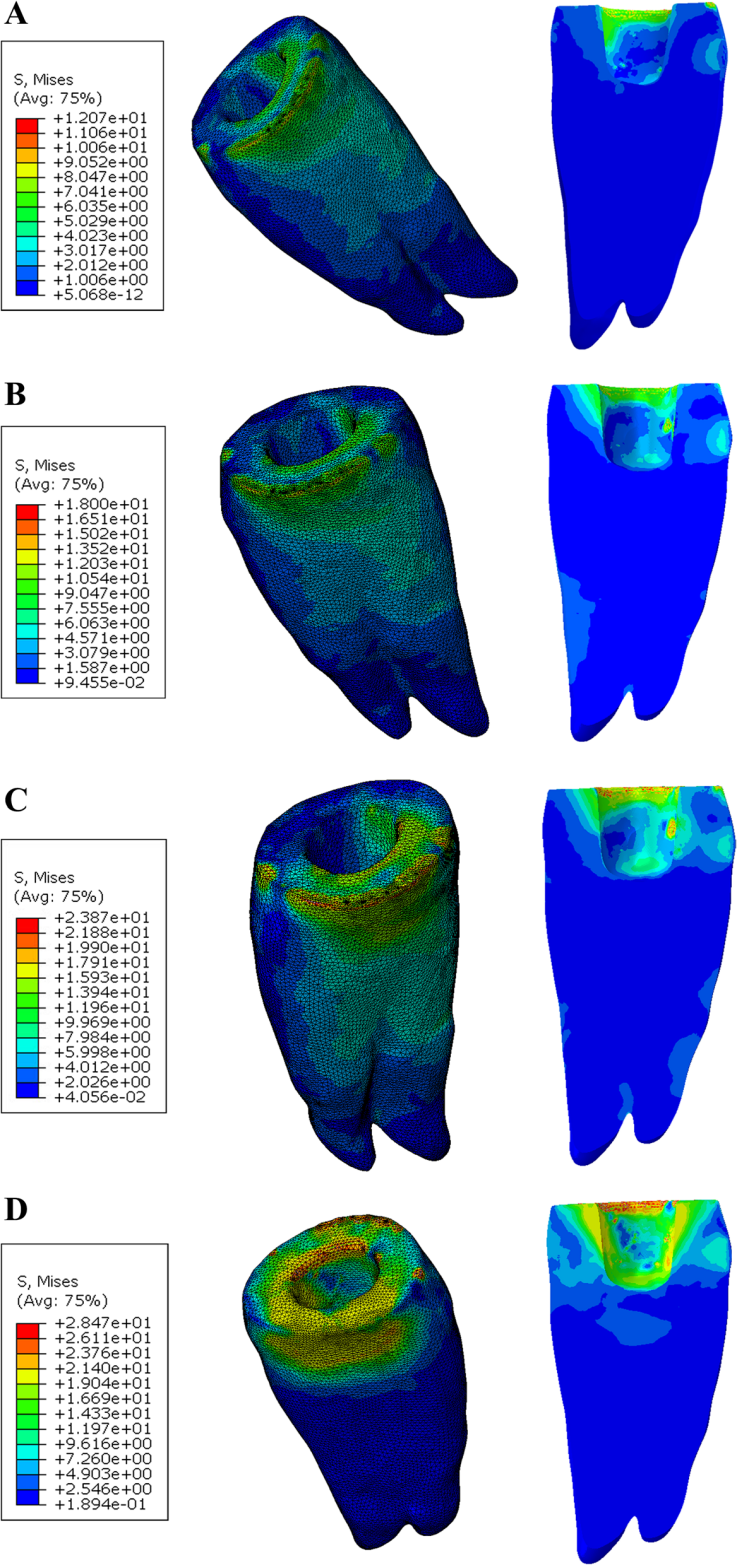
Von Mises stress distributions (MPa) in natural tooth (Butt joint design) **A** Restored with PEKK **B** Restored with Nacera Hyprid **C** Restored with Emax CAD **D** Restored with Zirconia

**Fig. 8:**
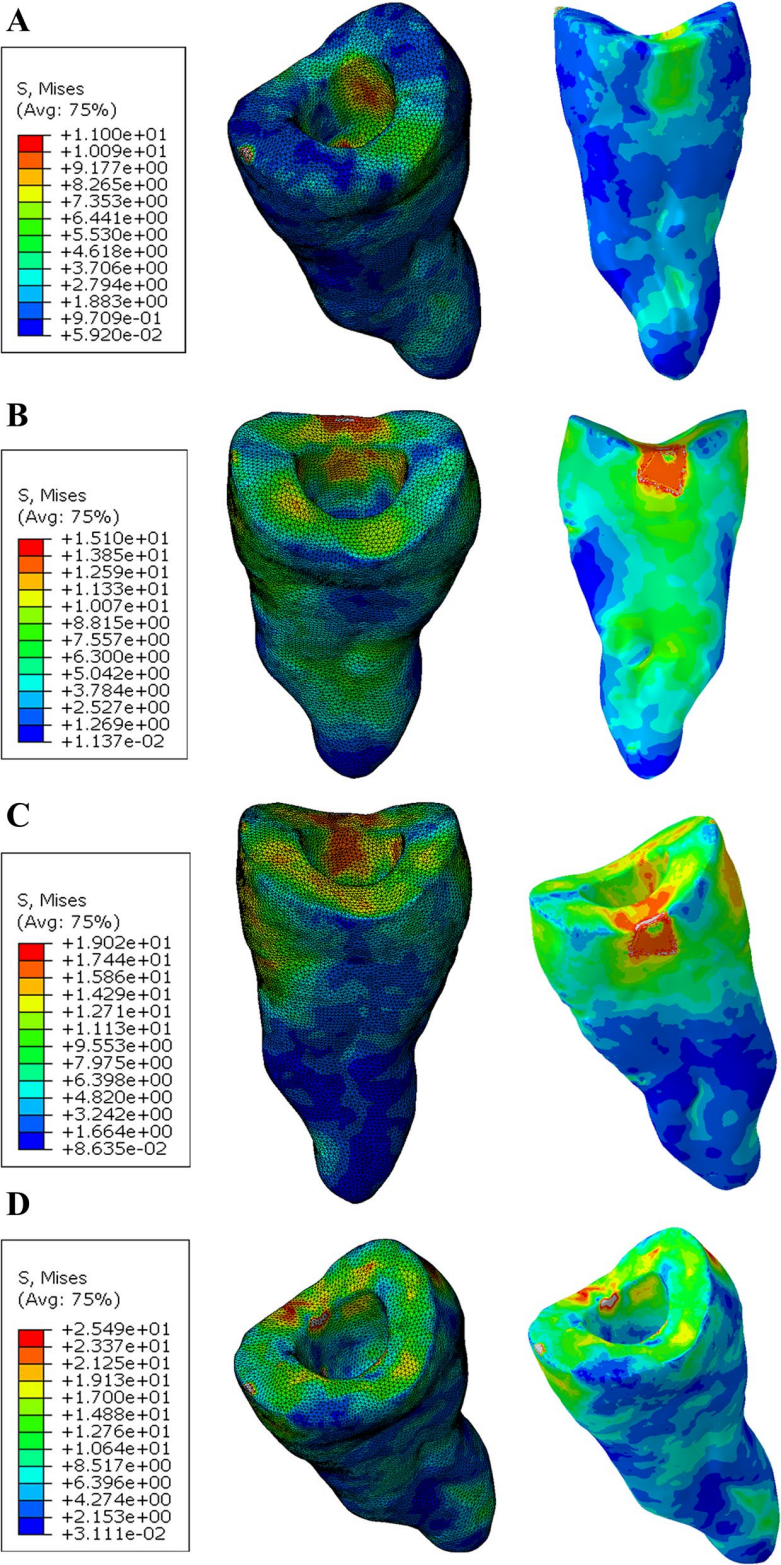
Von Mises stress distributions (MPa) in natural tooth (Anatomical design) **A** Restored with PEKK **B** Restored with Nacera Hyprid **C** Restored with Emax CAD **D** Restored with Zirconia

Modified von Mises (mvM) stress In the tooth, the group restored with PEKK showed slightly lower mvM stress values (11.1 MPa). In comparison, the Zirconia restorations presented higher (mvM) stress values (28.4 MPa), the lowest values of mvM stress were observed in restorations with anatomical occlusal reduction followed by restorations with butt joint reduction (A PE < B PE < A NH < B NH < A EM < B EM < A ZR < B ZR). According to the color map stress distribution there was a uniform distribution pattern. The highest stress values were concentrated at the cervical area (Figs. 7 and 8).

**Fig. 9:**
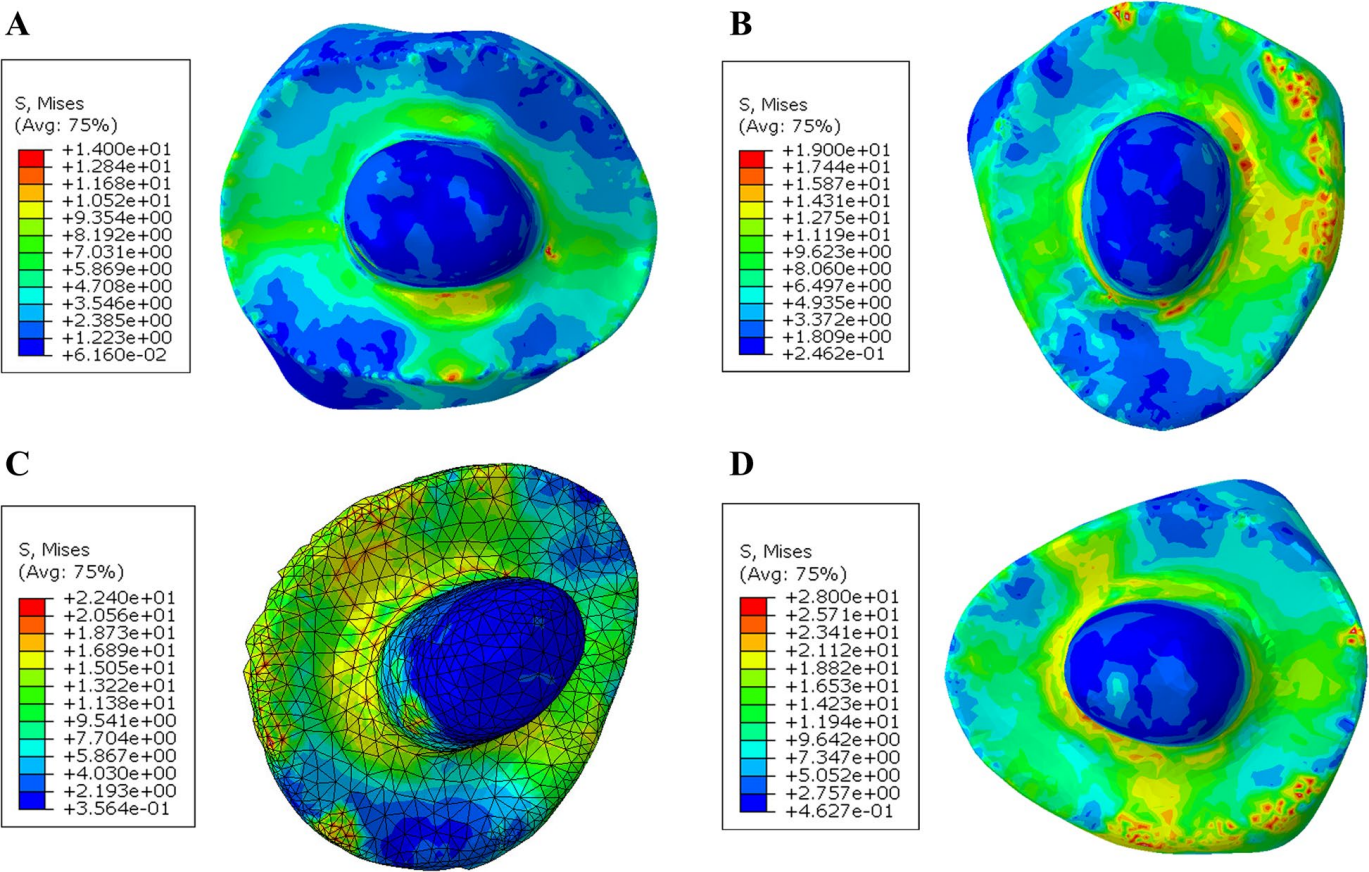
Von Mises stress distributions (MPa) in the cement lines between endocrown restoration and the tooth (Butt joint design)

**Fig. 10:**
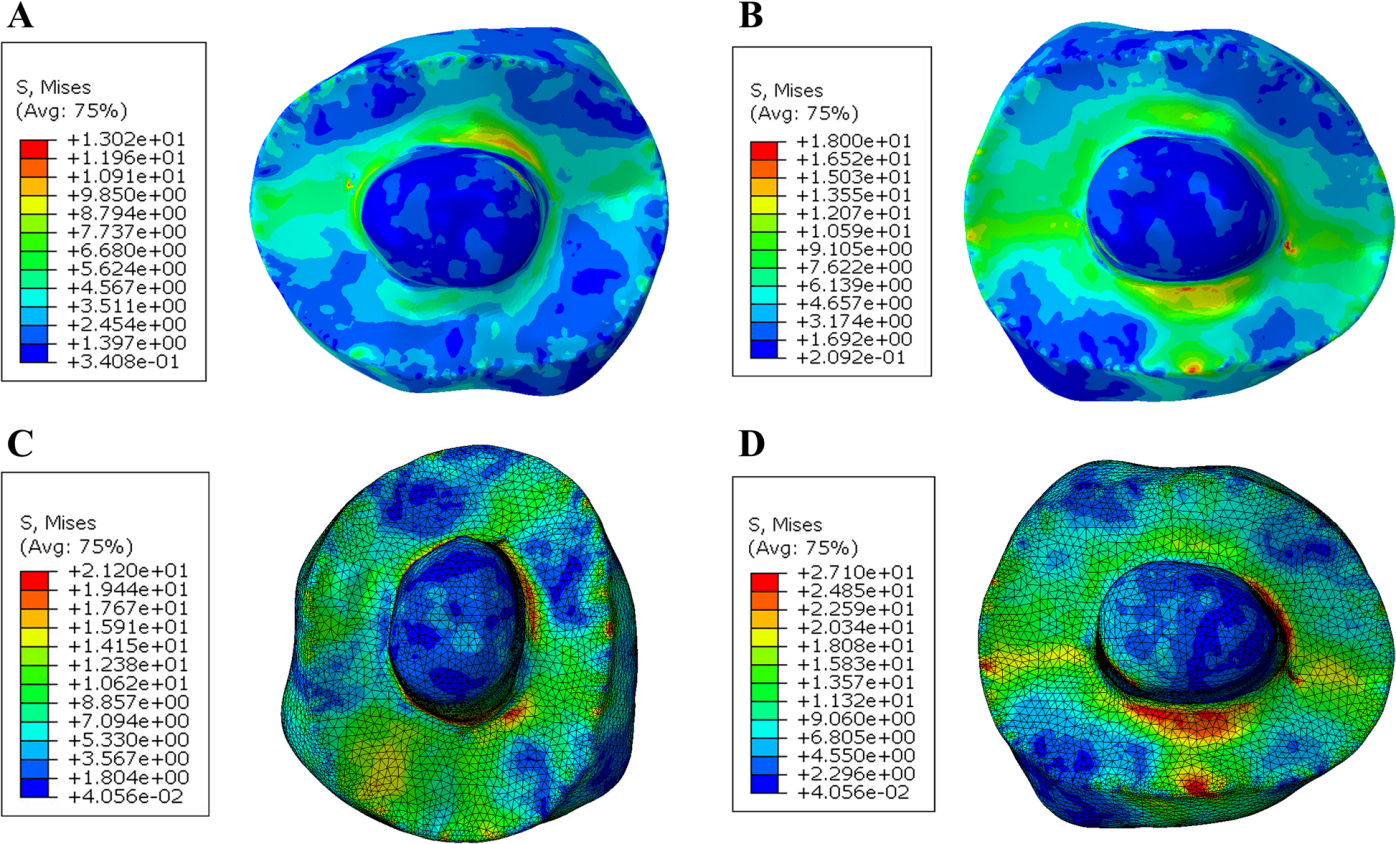
Von Mises stress distributions (MPa) in the cement lines between endocrown restoration and the tooth (Anatomical design)

The maximum mvM stress value and stress distribution in the cement line between the endocrown system and the tooth was in B PE model (28.1 MPa) and the lowest mvM stress values was in A ZR model (13.1 MPa) (Figs. 9 and 10).

**Fig. 11:**
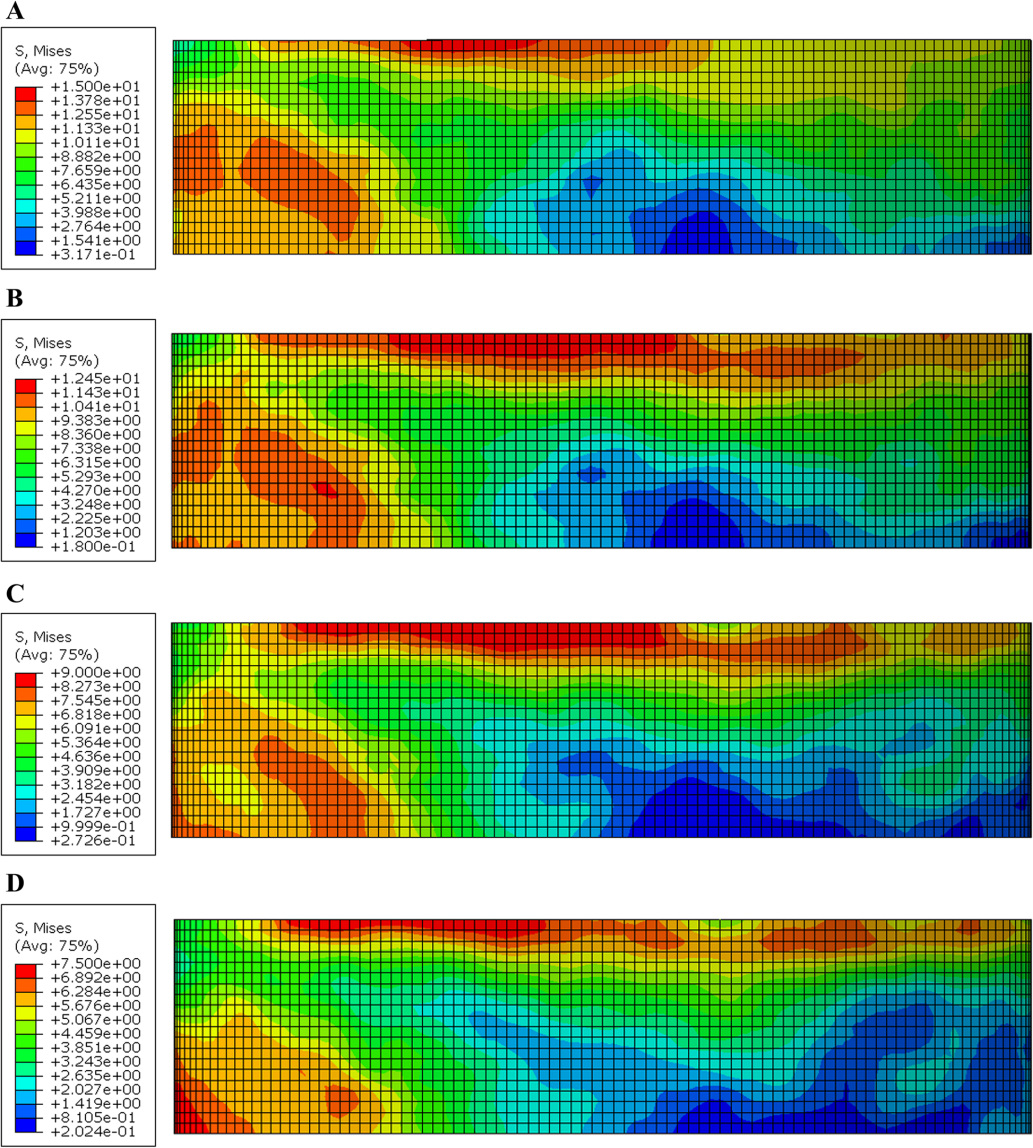
Von Mises stress distributions (MPa) in the flowable composite (Butt joint design) **A** Zirconia **B** Emax CAD **C** Nacera Hyprid **D** PEKK

**Fig. 12:**
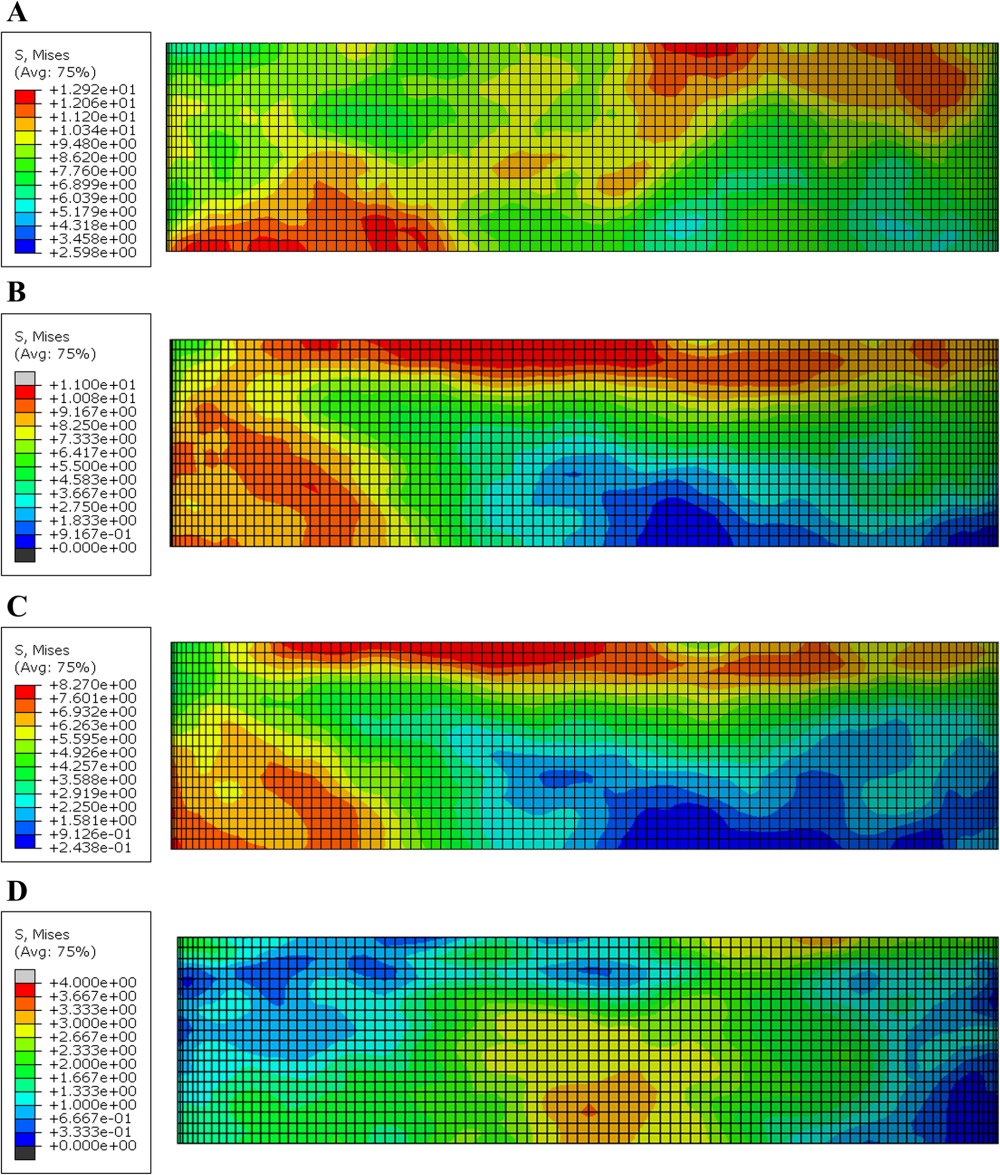
Von Mises stress distributions (MPa) in the flowable composite (Anatomical design) **A** Zirconia **B** Emax CAD **C** Nacera Hyprid **D** PEKK

The stress distribution in the flowable composite layer showing the maximum stress value in the model B ZR (15.1 MPa) and the less value in the model A PE (4.1 MPa). The mvM stress values of all models were less than the tensile stress of the flowable composite (36.50 MPa) [42]. According to the color maps for all models, the maximum stress concentration was determined at the top surface of composite (Figs. 11 and 12).

## Discussion

This study aimed to evaluate the influence of restorative material type and occlusal reduction on the fracture resistance and stress distribution patterns of endocrown restorations and teeth. The findings of this study revealed that all these factors significantly affected both the fracture resistance and stress distribution in both the restoration and the tooth. As a result, the null hypothesis was rejected.

The treatment of extensively damaged endodontically treated teeth continues to pose a challenge in dentistry [2]. Typically, these teeth are restored using standard post-retained restorations. However, with the advancements in CAD/CAM technology and adhesive techniques, conservative endocrowns have emerged as a reliable and favorable restorative approach [36]. Minimally invasive preparation, which aims to preserve as much tooth structure as possible, is considered the gold standard for tooth repair [36].

The selection of a suitable restorative material plays a crucial role in enhancing the effectiveness of an endocrown restoration. One commonly used restorative material, known for its long-term clinical performance in single unit crowns and endocrowns, is lithium disilicate-reinforced CAD/CAM ceramic material (IPS e.max CAD), which has an average flexural strength of 530 MPa [35]. It was chosen as the first material for endocrown restoration in this study. Glass ceramics offer several advantages over other ceramic materials, including biomimicry and aesthetic attributes that closely resemble natural teeth, thanks to their similar wear coefficient [46]. The second material used was a multilayer translucent zirconia (4YSZ) from the fourth generation of zirconia, with an average flexural strength of 750 MPa. It exhibits favorable mechanical and optical properties [44]. The third material, Nacera Hybrid, is a new hybrid ceramic material designed for long-term restorations. It has a composition of 50% nano-glass and 50% polymer matrix [39]. Nacera Hybrid does not require firing as it is already fully polymerized. It offers respectable aesthetics, a suitable level of flexibility, and adaptability. It can be used for both permanent restorations and long-term cosmetic temporaries [19]. In this study, PEKK was used as a coping material for endocrowns. It is considered a unique and appealing material for endocrown systems [22, 23].

The maxillary first premolars were selected for this research as there is still uncertainty regarding the successful restoration of endodontically treated premolars using endocrowns. Previous investigations have generated controversy regarding the use of endocrowns as a potential treatment for endodontically treated premolars [10, 32, 35]. These premolars were chosen to assess the effectiveness of various endocrown designs in restoring teeth with unique anatomy and distinctive morphology, which are prone to cusp deflection [45] and fracture under occlusal loads [24]. Full anatomy endocrowns were used to restore the premolar teeth as they are believed to function more similarly to the clinical setting compared to ceramic discs, according to some reports [42]. Flexural strength and fracture resistance are commonly used terms to evaluate material strength and predict its success in clinical applications [7]. In this study, self-cured epoxy resin was used to fix the selected teeth due to its elastic modulus (12GPa), which is similar to that of the alveolar bone (18GPa). By creating a periodontal ligament (PDL) layer around the roots in the model, it could act as a shock absorber, allowing for accurate tooth movement modeling and uniform stress distribution in the synthetic PDL material [22].

Similar to the in-vitro investigation, the fracture load was applied axially rather than laterally in order to assess stress concentration and the risk of failure in the endocrown systems used in this study. Axial loading allows for the evaluation of the influence of inherent material properties (modulus of elasticity) and the thickness of the restorative materials on their mechanical performance, while lateral loading would primarily assess the effect of bonding and adhesion of the endocrown [22, 33]. Various techniques were employed for stress analysis to estimate different strains on oral tissues and predict the performance of restorative materials in clinical settings. Finite Element Analysis (FEA) has been widely used in studies on dental biomechanics [10, 22, 33]. The von Mises stresses, which encompass tensile, compressive, and shear stresses from the complete stress field, are considered a reliable indicator of the risk of failure for brittle materials.

The mean values of fracture loads detected in all examined groups under axial loading exceeded the biting force in the maxillary premolar region (normal = 450 N or during clenching = 660 N). These values also surpassed the highest human masticatory forces reported in the literature (850 N in the posterior area and 900–1000 N in the first molar region with severe parafunctional bruxing habits) [46, 47]. After the artificial thermomechanical aging technique, all studied specimens survived without exhibiting any indicative signs of early failure. This finding suggests that all groups are capable of withstanding the repetitive occlusal loads typically experienced during oral function.

When comparing the fracture resistance values of different CAD/CAM ceramic endocrowns, one-way ANOVAs of the significant two-way interactions revealed that PEKK endocrowns samples made with anatomical preparation (1862.0 ± 137) exhibited significantly higher values than all other materials (Zirconia, E max CAD, and Nacera Hybrid endocrowns). This polymer material (PEKK), which possesses mechanical qualities similar to those of natural dentition. Its compressive strength (246 MPa) is similar to that of dentin (297 MPa), creates a better biomechanical fit between the tooth and the restoration, thereby increasing the reliability of the restorative system [13, 22]. Zirconia endocrowns demonstrated higher fracture strength resistance values compared to E max and Nacera endocrowns. This finding can be attributed to its high flexural strength and fracture toughness, which result from its composition primarily consisting of crystalline particles, making it suitable for lengthy restorations.

Additionally, a comprehensive review emphasized the significant impact of the polymer matrix on force and stress distribution in teeth, supporting the use of hybrid ceramics for reconstructing maxillary premolars. Several studies have employed resin ceramics for restoring maxillary premolars due to their better fracture strength and decreased risk of catastrophic failure. Furthermore, a comprehensive review by Al-Dabbagh et al. [7] highlighted the significant impact of the polymer matrix on force and stress distribution in teeth. This supports our hypothesis of accepting hybrid ceramics for reconstructing maxillary premolars. Numerous studies [10, 31, 33, 37] have employed resin ceramics to restore maxillary premolars, as these materials have demonstrated better fracture strength and a decreased risk of catastrophic failure.

It appears that the use of anatomical occlusal preparation in PEKK endocrowns resulted in higher fracture resistance values compared to butt preparation. This can be attributed to improved stress and force distribution that adheres to the natural premolar anatomy. The anatomical preparation allowed for a consistent thickness across the entire occlusal surface, which helps to avoid stress concentration and potential fracture points. These findings are consistent with the study conducted by Kalay et al., who concluded that a minimal anatomical cusp reduction of 2.5 mm resulted in better resistance to fracture and a favorable mode of failure in endodontically treated maxillary premolars [12]. Additionally, Foad et al. found that anatomical cusps endocrown preparation significantly increased fracture resistance in upper premolars [32]. The use of anatomical contour in occlusal preparation provides better force distribution along the major tooth axis, contributing to improved stability and resistance to compressive pressures [44].

In the present study, different failure modes were observed depending on the type of endocrown system used. PEKK and Nacera Hybrid endocrowns with various occlusal designs demonstrated excellent performance compared to other ceramic materials. However, Emax and Zirconia endocrowns exhibited catastrophic fractures. This can be attributed to the different elastic moduli of these materials which allows for bending and stress absorption. Conserva [48] concluded that composite materials with a young’s modulus similar to dentine appear to be a favorable choice for reconstructing endodontically treated teeth. On the other hand, Zirconia and glass ceramics are rigid materials with high stiffness, leading to stress accumulation in the remaining tooth structure and catastrophic failures [13, 24]. A study conducted by Dartora et al. in 2021 [37] also concluded that monolithic zirconia had a higher rate of catastrophic fracture of the tooth structure. However, it is worth noting that these catastrophic failures typically occurred under loads that even patients with bruxism would not normally reach.

The present study revealed that different endocrown restoration materials have influenced the pattern of stress distribution on maxillary first premolar teeth that have undergone endodontic treatment. PEKK endocrowns demonstrated a better pattern of stress distribution compared to other endocrown restorations, followed by Nacera Hybrid, Emax CAD, and Zirconia. PEKK endocrowns effectively transferred reduced stresses to the remaining tooth structure, highlighting their tooth-friendly nature. These findings are consistent with a study by Zhu et al. [49], who reported that as the Young’s modulus of elasticity of the endocrown material increased, the von Mises stresses also increased in the remaining tooth structure, potentially increasing the likelihood of future tooth fracture. In contrast, Dejak and Młotkowski (2020) [40] reported that as the stiffness of the restorative material increased, lower von Mises stresses were transferred to the remaining tooth structure. Hybrid materials with a dual ceramic-polymer structure, such as Nacera Hybrid, exhibit integrated fracture propagation, homogeneous stress distribution, and shock absorption capabilities. Our findings support the concept of biomimetics, where substrates with comparable elasticity to tooth structure consistently and integrally respond to stress application, in contrast to materials with varying elasticity that behave inconsistently and are more prone to failure [24].

In terms of stresses produced in the endocrown systems, the PEKK restorative endocrown material exhibited the highest maximum stress value, followed by Zirconia, E.max CAD, and Nacera Hybrid. As the Young’s modulus of elasticity for PEKK is 5.1 GPa, for Nacera Hybrid is 9.9 GPa, and for dentin is 18.6 GPa. The compressive strengths of PEKK are 246 MPa and 297 MPa for dentin. On the other hand, E.max CAD and Zirconia endocrowns have high elastic moduli of 95 GPa and 210 GPa, respectively. Zirconia, being less elastic, is unable to follow the normal flexural movements of the tooth [24, 50]. This lack of elasticity creates zones of shear and tension at the interface between the cemented restoration and enamel or dentin. The amount of stress concentration is determined by the relative rigidity differences between the tooth and the cemented endocrown [13, 33].

In the literature, the luting cement is recognized as part of the dental tissues [22]. According to von Mises analysis, the stress value in the cement line was found to be lower in the Zirconia endocrown system and the tooth. This result is consistent with the FEA study conducted by Dejak and Młotkowski [40], who stated that as the stiffness of the restorative material increases, the stress transferred to the interface between the tooth and the material decreases. This result is also supported by Tribst et al. [51], who concluded that stresses in the cement layer were decreased between zirconia restorations and the tooth, but increased between leucite ceramic and the tooth. In other words, when the endocrown material has a low Young’s modulus of elasticity, the risk of catastrophic failure is reduced. Therefore, the overall stability of the system in the tooth, when utilizing a material with low elasticity for endocrown restoration, may depend on the elastic modulus and strength of the cement [33, 52, 53].

Indeed, this present study has certain limitations that should be acknowledged. Firstly, the sample size was relatively small, with only eight specimens used in each group. A larger sample size would provide more robust and reliable results. Furthermore, it is recommended to explore the effects of different methods of artificial aging on the fracture resistance of endocrown systems. This would allow for a more comprehensive understanding of the long-term performance and durability of these restorations. Additionally, the study only evaluated the effect of load application in the axial direction.

## Conclusions

Based on the findings of this in-vitro and FEA analysis, the following conclusions were drawn:

1. All endocrown systems evaluated in the study are capable of withstanding the normal occlusal forces experienced during chewing.
2. PEKK endocrowns demonstrated higher fracture resistance compared to other ceramic endocrowns. This suggests that PEKK is a promising material for endocrown restorations, as it can withstand higher forces without fracturing.
3. PEKK endocrowns also exhibited a better stress distribution pattern on the teeth compared to other ceramic endocrowns. This indicates that PEKK endocrowns can distribute occlusal forces more evenly, reducing the risk of stress concentration and potential failure.
4. Anatomical occlusal design significantly enhanced the fracture resistance of endocrowns, particularly in maxillary premolars. This suggests that preserving the natural tooth anatomy and contour during the preparation of endocrowns can improve their overall strength and longevity.

These conclusions highlight the potential benefits of using PEKK material and anatomical occlusal design in endocrown restorations. However, it is important to note that these findings are based on in-vitro and FEA analysis, and further clinical studies are needed to validate these results in a real-world setting.

## Data Availability

The data and materials were available.
